# Supplementary material for: COVID-19 Patients’ Medication Management during Transition of Care from Hospital to Virtual Care: A Cross-Sectional Survey and Audit
Source: Pharmacy (Basel). 2023 Sep 28;11(5):157. doi: 10.3390/pharmacy11050157 (PMC10610024; doi:10.3390/pharmacy11050157)
Supplement: Supplementary file 1 [file pharmacy-11-00157-s001.zip › COVID Model of Care Patient survey.pdf]

Evaluation of medication management and safety at transition of care from hospital of COVID-19 patients: a process evaluation

Patient telephone survey

Introduction:

- Person delivering the telephone survey to introduce themselves
- Explain objectives of the survey using the script below:

Hello, my name is…

As a patient of GCHHS, we value your feedback as we are constantly seeking new ways to improve our service.

We have recently implemented new services to provide support to patients with COVID-19 in the community. We would like to invite you to take part in a short telephone survey so we can gain some feedback on your experiences. The survey will only take about 5 minutes and your feedback will really assist us in determining what we are doing well and what requires improvement.

Your feedback will remain anonymous and will not impact on your relationship with GCHHS. The telephone survey is voluntary and you are able to withdraw your involvement at any time.

These new models of care we have implemented at GCHHS to manage the COVID-19 surge forms part of a study we are conducting about medication safety and management of patients with COVID -19 who are managed by GCHHS whilst not in hospital. We are contacting patients who were inpatients in a hospital ward or inpatient unit and transferred to a virtual ward (state here the specific model the patient is in to clarify).

This study has been approved by the GCHHS ethics committee.

Do you agree to participate?

Yes ☐ No ☐

Date of consent: __________________________ Time of consent: _________am/pm

If yes: Do you have any questions before we start?

Name of person obtaining consent: ____

Note for interviewer to adapt terminology of 'virtual ward' depending on where the patient was transferred to either:

- **Virtual Ward**
- **Hospital In The Home**
- **Hospital In The Hotel**

QUESTIONS

Support received from GCHHS when transferred

1. Did one of the staff members talk to you about how to use your medicines when you left the hospital ward?

   ☐ Yes ☐ No ☐ Unsure

   1a If yes, which staff member was it? This could be more than one staff member, let me know about all of them.

   ☐ Pharmacist ☐ Doctor ☐ Nurse ☐ Other ☐ Don't know

2. Did you receive a written list of your medicines with instructions on how to use them when you left the hospital ward?

   ☐ Yes ☐ No ☐ Unsure

   2a If yes, was the list useful?

   ☐ Yes ☐ No ☐ Unsure

3. Did your medicines change whilst you were in hospital?

   ☐ Yes ☐ No ☐ Unsure

   3a If yes, can you provide more details.

4. Do you believe that the information you received from the hospital about your medicine when you were transferred was adequate for you to know how to take your medicine?

   ☐ Yes ☐ No ☐ Unsure

   4a Could you provide me with more details of how the hospital staff could have provided you with more information?

   ____

The next few questions are about you and your medicines whilst in the [1]virtual ward:

5. Did you have enough supply of your medicines whist in the [1]virtual ward?

   ☐ Yes ☐ No ☐ Unsure

   5a If no, which of your medicines did you not have with you?

6. Did you receive advice about how to take your medicines while in the [1]virtual ward?

☐ Yes ☐ No ☐ Unsure

6a If yes, who provided the advice? This could be more than one staff member, let me know about all of them.

☐ Pharmacist ☐ Doctor ☐ Nurse ☐ Other ☐ Don't know

7. Please rate the support received from the hospital about how to take your medicines while in the [1]virtual ward on a scale of one to five (1-5) where:

☐ 1. Very poor ☐ 2. Poor ☐ 3. Fair ☐ 4. Good ☐ 5. Very good

8. How often were you contacted about your medicines while in the [1]virtual ward?

☐ More than once per day ☐ Daily ☐ Every 2nd day

☐ Only when I was transferred ☐ Other, please provide details ____________________

9. Please rate how confident you were in how to use your medicines whilst in the [1]virtual ward on a scale of one to five (1-5) where:

☐ 1. Not at all confident ☐ 2. Slightly confident ☐ 3. Somewhat confident

☐ 4. Moderately confident ☐ 5. Extremely confident

10. Please rate how concerned you were about taking your medicine correctly as prescribed by the hospital?

☐ 1. Not at all concerned ☐ 2. Slightly concerned ☐ 3. Somewhat concerned

☐ 4. Moderately concerned ☐ 5. Extremely concerned

11. Did you have opportunity to ask questions about your medicines whilst in the [1]virtual ward?

☐ Yes ☐ No ☐ Unsure

---

[1] Use appropriate depending one where patient was transferred to: Virtual Ward, Hospital In The Home, Hospital In The Hotel

12. Do you have a special way to help you remember to take your medicines?

☐ Yes ☐ No ☐ Unsure

12a Could you provide me with more details of what you do to remember to usually take your medicines?

13. Did you miss any medicine doses whilst in the [1]virtual ward?

☐ Yes ☐ No ☐ Unsure

12a If yes, which medicine(s)

12b How often did this happen?

14. Did you take more medicine doses than prescribed whilst in the [1]virtual ward?

☐ Yes ☐ No ☐ Unsure

13a If yes, which medicine(s)

13b How often did this happen?

15. Was there a time you wanted to speak to a pharmacist about your medicines while you were in the [1]virtual ward but failed to make contact?

☐ Yes ☐ No ☐ Unsure

16. Did you know how to contact a pharmacist for assistance while you were in the [1]virtual ward if you needed support?

☐ Yes ☐ No ☐ Unsure

Optional: Open-ended questions

17. Could you provide me with some overall comments about the level of support you received regarding how to take your medicines whilst you were in the [1]virtual ward?

    ____

18. How can GCHHS improve the medicine support we provide to patients in the [1]virtual ward?

    ____

19. Do you have any other comments?

    ____

END OF SURVEY – Thank the patient for their participation
